# Ultrafine particles affect the balance of endogenous pro- and anti-inflammatory lipid mediators in the lung: *in-vitro* and *in-vivo* studies

**DOI:** 10.1186/1743-8977-9-27

**Published:** 2012-07-18

**Authors:** Ingrid Beck-Speier, Erwin Karg, Heidrun Behrendt, Tobias Stoeger, Francesca Alessandrini

**Affiliations:** 1 Comprehensive Pneumology Center, Institute of Lung Biology and Disease, Helmholtz Zentrum München, German Research Center for Environmental Health, Neuherberg, Germany; 2 Cooperation Group ,,Comprehensive Molecular Analytics" (CMA), Helmholtz Zentrum München, German Research Center for Environmental Health, Neuherberg, Germany; 3 Institute of Allergy Research, Helmholtz Zentrum/Technische Universität München, Helmholtz Zentrum München, German Research Center for Environmental Health (GmbH), ZAUM Center for Allergy and Environment, Neuherberg and Munich, Germany; 4 Christine Kuehne Center for Allergy Research & Education (CK-CARE), Munich, Germany; 5 Focus-Network Nanoparticles and Health (NanoHealth), Helmholtz Zentrum München, German Research Center for Environmental Health (GmbH), Neuherberg, Germany

**Keywords:** Lipid mediators, Particulate matter, Inflammation, Allergy

## Abstract

**Background:**

Exposure to ultrafine particles exerts diverse harmful effects including aggravation of pulmonary diseases like asthma. Recently we demonstrated in a mouse model for allergic airway inflammation that particle-derived oxidative stress plays a crucial role during augmentation of allergen-induced lung inflammation by ultrafine carbon particle (UfCP) inhalation. The mechanisms how particle inhalation might change the inflammatory balance in the lungs, leading to accelerated inflammatory reactions, remain unclear. Lipid mediators, known to be immediately generated in response to tissue injury, might be strong candidates for priming this particle-triggered change of the inflammatory balance.

**Methods:**

We hypothesize that inhalation of UfCP may disturb the balance of pro- and anti-inflammatory lipid mediators in: i) a model for acute allergic pulmonary inflammation, exposing mice for 24 h before allergen challenge to UfCP inhalation (51.7 nm, 507 μg/m^3^), and ii) an *in-vitro* model with primary rat alveolar macrophages (AM) incubated with UfCP (10 μg/1 x 10^6^ cells/ml) for 1 h. Lungs and AM were analysed for pro- and anti-inflammatory lipid mediators, namely leukotriene B_4_ (LTB_4_), prostaglandin E_2_ (PGE_2_), 15(S)-hydroxy-eicosatetraenoic acid (15(S)-HETE), lipoxin A_4_ (LXA_4_) and oxidative stress marker 8-isoprostane by enzyme immunoassays and immunohistochemistry.

**Results:**

In non-sensitized mice UfCP exposure induced a light non-significant increase of all lipid mediators. Similarly but significantly in rat AM all lipid mediators were induced already within 1 h of UfCP stimulation. Also sensitized and challenge mice exposed to filtered air showed a partially significant increase in all lipid mediators. In sensitized and challenged mice UfCP exposure induced highest significant levels of all lipid mediators in the lungs together with the peak of allergic airway inflammation on day 7 after UfCP inhalation. The levels of LTB_4_, 8-isoprostane and PGE_2_ were significantly increased also one day after UfCP exposure. Immunohistochemistry localized highest concentrations of PGE_2_ especially in AM one day after UfCP exposure.

**Conclusion:**

Our results suggest that UfCP exposure affects the balance between pro- and anti-inflammatory lipid mediators. In allergic mice, where the endogenous balance of pro- and anti-inflammatory mediators is already altered, UfCP exposure aggravates the inflammation and the increase in anti-inflammatory, pro-resolving lipid mediators is insufficient to counterbalance the extensive inflammatory response. This may be a contributing mechanism that explains the increased susceptibility of asthmatic patients towards particle exposure.

## Background

Lipid mediators are involved in the onset, progression and resolution of acute inflammation [1]. Tissue injury, allergens or microbes activate formation and release of arachidonic acid-derived lipid mediators, such as prostaglandins and leukotrienes, which regulate early events in the inflammatory response. Those include changes of blood flow, oedema and leukocyte recruitment. A key feature of bioactive lipid mediators is the rapidity with which they are synthesized; they appear within minutes upon stimulation, as opposed to peptide mediators such as cytokines, which require hours to be generated. By their specific receptors they mediate biological and physiological activities and gene expression for a variety of inflammatory events [2-5]. At a later time point counter-regulatory lipid mediators such as lipoxins, resolvins and protectins, are generated to initiate the resolution of inflammation [6]. The resolution of acute lung inflammation is an active process and not merely characterized by the absence of pro-inflammatory signals. The restoration of homeostasis is coordinated by specific mediators and cellular events. In response to inflammatory stimuli and injury, infiltrating leukocytes and tissue-resident cells interact to generate lipoxins, which are involved in the termination of inflammation [7].

Allergic asthma is a complex disorder of the conducting airways involving airway inflammation, declining airway function and hyperresponsiveness [8]. The prevalence of asthma has rapidly increased over the last few decades to epidemic proportions and is expected to rise dramatically over the next 15–20 years [9,10]. Asthma is thought to arise from a complex interplay of genetic susceptibility and environmental factors. Epidemiological and clinical studies have shown an association between increased ambient airborne particle concentrations and adverse respiratory health effects, leading to exacerbations of respiratory morbidity [11-13]. In particular, there is growing evidence that ultrafine ambient particles play an important role in the pathogenesis of respiratory diseases [14-16]. Ultrafine particles (< 0.1 μm aerodynamic diameter) are characterized by a high number concentration, low mass concentration and a large surface area per mass [17]. Due to their small size they have a higher deposition rate in the peripheral lung compared to fine particles (< 2.5 μm aerodynamic diameter) [18,19]. Primary ultrafine particles are formed during gas-to-particle conversion or during incomplete fuel combustion processes [20].

Ultrafine carbon particles (UfCP) exert an adjuvant effect for allergen-induced lung inflammation [21]. Recently, we reported that the inhalation of UfCP induced augmentation of oxidative stress in the lungs of a mouse model for asthma in addition to lung inflammation, activation of nuclear factor κB, cytokine release and airway hyperreactivity [22]. In this follow up study we hypothesize that UfCP may affect the balance between endogenous pro- and anti-inflammatory and pro-resolving arachidonic acid-derived lipid mediators. We therefore evaluated concentrations of pro-inflammatory LTB_4_, immune-modulating PGE_2_, anti-inflammatory 15(S)-hydroxytetraenoic acid (15(S)-HETE), pro-resolving lipoxin A_4_ (LXA_4_) and 8-isoprostane as marker for oxidative stress in two different models: i) an *in-vivo* study with healthy mice and in an allergic mouse model; and ii) an *in-vitro* model with primary alveolar macrophages (AM) of healthy rats as part of the primary pulmonary defence system. We quantified the endogenous concentration of lipid mediators in homogenates of AM and lung tissue and determined immunohistochemically the localisation of two of the mediators, PGE_2_ and 8-isoprostane, in lung tissue and cells.

## Materials and methods

### Materials

Phosphate-buffered saline (PBS) with and without Ca^2+^/Mg^2+^ was purchased from Biochrome (Berlin, Germany); RPMI was from PAA Laboratories (Linz, Austria); fetal calf serum, penicillin, streptomycin and amphotericin were from Life Technologies (Eggenstein, Germany); HEPES (2-(4-(2-hydroxyethyl)-1-piperaziyl)-ethansulfonic acid) from Sigma-Aldrich Chemie (Munich, Germany); all other chemicals (analytical or HPLC grade) were from Merck (Darmstadt, Germany).

### Ultrafine carbon particles (UfCP) exposure

Airborne UfCP were generated by electric spark discharge (model GFG 1000, Palas, Germany) providing aggregated carbon particles as previously described [21,23,24]. UfCP average size distribution was characterized by a count median diameter of 51.7 nm, an average mass median diameter of 84.7 nm and a geometric standard deviation of 1.54. Temporal variability was below 6.3% for all size distribution parameters. UfCP exposures used for the *in-vivo* study were performed in 330 liter whole body exposure chambers with an air exchange rate of 18.2 h^-1^ (100 lpm at an average displacement velocity of 5 mm s^-1^) for 24 h.

Particle mass concentration was 507 μg m^-3^ (± 26) μg/m^3^, which corresponds to an average particle number concentration of 9.3 · 10^6^ cm^-3^ (± 0.6) cm^-3^. Temporal variability was below 5.1% for both number and mass concentration. For the *in-vitro* study filter samples of the UfCP (10 μg/ml) were suspended in liquid by ultrasonic stirring and incubated with the cells for 60 min as described later in more detail.

### Animals

Five to seven-week-old female Balb/c mice (Charles River, Sulzfeld, Germany) and twelve- to −14-wk-old, male Wistar Kyoto rats (WKY/Kyo@Rj; Janvier, France) were housed in a humidity- (55% relative humidity) and temperature- (22°C) controlled room in individually ventilated cages (Ventirack^TM^, cage type CU-31), maintained on a 12-h day/night cycle prior to the study. Laboratory animal diet and water was provided *ad libitum*. The studies were conducted under federal guidelines for the use and care of laboratory animals and were approved by the Government of the District of Upper Bavaria and by the Helmholtz Zentrum Institutional Animal Care and Use Committee.

### In vivo study with allergic mice

#### Allergen sensitization/challenge protocol

The animal study has been conducted as described previously [22]. In brief, BALB/c mice were sensitized by repetitive intraperitoneal injections of 1 μg OVA (Sigma, grade VI)/Alum (Pierce, 2.5 mg) in phosphate buffered saline (PBS with Ca^2+^/Mg^2+^) on days 0, 14, 28, 48, 72. Blood samples were taken before and after sensitization. OVA-specific IgE and IgG1 were measured in plasma samples by ELISA as described previously [21]. OVA/albumin sensitized mice, compared to non sensitized controls, were characterized by high titers of OVA-specific IgE (31.7 ± 3.3 vs 0.1 ± 0.01 μg/ml) and OVA-specific IgG1 (1178.4 ± 211.2 vs <0.1 μg/ml). On day 86 the mice were aerosol-challenged for 20 min with 1% OVA in PBS or with PBS alone delivered by Pari-Boy nebulizer (Pari GmbH, Starnberg, Germany). In order to minimize LPS contamination, the OVA solution prepared for aerosol challenges was eluted over polymyxin B columns (Endotoxin Detoxi-Gel^TM^, Pierce Chemical Co., Rockfort, IL, USA) according to the manufacturer’s instruction.

#### Study design

The mice were divided into 4 experimental groups: non sensitized mice exposed to UfCP (UfCP group), OVA sensitized mice exposed to filtered air 24 h prior to OVA challenge (S/OVA group), OVA sensitized mice exposed to UfCP 24 h prior to allergen challenge (S/UfCP/OVA group), and age-matched untreated animals served as baseline controls (untreated). Zero, 1, or 7 days after OVA challenge or after termination of UfCP exposure, BAL was performed as previously described [21]. After BAL the right lobes of the lungs were snap frozen in liquid nitrogen and stored at −80°C for subsequent analysis (n = 4-9/time point). The left lobes of 2 lungs/time point/group were fixed in 10% buffered formalin for immunohistochemistry.

### In vitro study with primary rat alveolar macrophages (AM)

#### Alveolar macrophages

AM from healthy WKY rats were isolated by bronchoalveolar lavage (BAL, repeated 5 times) using fresh aliquots of Ca^2+^/Mg^2+^-free PBS kept at 37°C. After 20 min centrifugation at 400 x *g* cells were resuspended in RPMI medium containing penicillin (100 U/ml), streptomycin (100 U/ml), amphotericin (2.5 μg/ml) and 5% fetal calf serum (further referred as RPMI medium). Viability was about 97% as determined by trypan blue exclusion. Microscopic examination after May Grünwald Giemsa staining of cytospin preparations identified 95–100% of the cells as AM.

#### Incubation of alveolar macrophages with particles

AM were incubated in RPMI medium with UfCP at a mass concentration of 10 μg/1x10^6^ AM/ml for 60 min at 37°C, corresponding to a specific surface area of 75 cm^2^/1x10^6^ AM/ml [23]. Control cells were incubated in parallel without particles. UfCP were prepared by electric spark discharge (model GFG1000, Palas, Germany), suspended in distilled water by repeated vortexing and sonification [23] providing agglomerated carbon particles as previously described [23,24]. After incubation, the cells were harvested by centrifugation 400 x *g* for 10 min at room temperature. The cell pellets were resuspended in half of the incubation volume with cold HEPES, pH 7.4, and homogenized by sonification (3 times, 15 s each time) in ice. The cell homogenates were centrifuged at 10,000 x *g* for 15 min at 4°C. The supernatants were taken for measurements of protein and lipid mediators [25,26]. Analysis of the cell lysates is a reliable tool to quantify the parameters in each sample [25].

### Analysis

#### Lipid mediators and protein determination

For analysis of lipid mediators, the supernatants of cell or lung homogenates were used. Lung homogenates (about 50 mg lung tissue/500 μl) were prepared by disrupting lung tissue in HEPES, pH 7.4, with lysing matrix E (FastPrep FP120 cell disrupter; MP Biomedicals Germany GmbH, Eschwege, Germany). After centrifugation (3200 x *g* at 4°C for 10 min), aliquots of the obtained supernatants were taken for determination of protein and lipid mediators. Aliquots of supernatants derived from cell homogenates of particle-treated cells (prepared as described above) and those from lung homogenates were deproteinized by adding 8-fold volume of 90% methanol containing 0.5 mM EDTA and 1 mM 4-hydroxy-2,2,6,6-tetramethylpiperidine-1-oxyl, pH 7.4 [23]. These methanol suspensions were stored at −40°C for 24 h followed by two centrifugation steps at 10,000 x *g* for 20 min at 4°C with a 24 h interval to remove the proteins. Aliquots of the obtained supernatants were dried in a vacuum centrifuge, dissolved in assay buffer, and used for quantification of PGE_2_, LTB_4_ 15(S)-HETE, 8-isoprostane and LXA_4_ by their specific enzyme immunoassays (Cayman Chemical Company, Ann Arbor, MI, USA) according to the instructions of the manufacturer. Protein was measured at 595 nm using 5 μl of homogenate and 200 μl of 1:5 diluted Biorad solution (Bio-Rad, Munich, Germany) with bovine serum albumin as standard.

#### Immunohistochemistry

After formalin fixation and paraffin embedding, 5 μm sections of the lungs obtained on day 1 and 7 were cut. After deparaffinization the sections were placed in 0.1 M citric acid, pH 6.0, and heated in a microwave at low power for 15 min; immunostaining was performed using an ABC-based method (Vector Laboratories, Burlingame, CA) as previously described [27]. As primary antibodies, α-8-isoprostane and α-prostaglandin E_2_ were used (Oxford Biomedical Research, Oxford, MI, USA). Negative controls included use of buffer alone or respective dilutions of non-specific purified goat and rabbit immunoglobulin G (IgG) in the primary layer (Vector Laboratories).

#### Statistics

Statistical significance was determined by one-way analysis of variance with post-hoc Bonferroni test for the *in-vivo* study and by analysis of variance and two-sample Student`s *t*-test for the *in-vitro* study (STAT-SAK, version2.12, by G. E, Dallal, 1986; Malden, MA). Changes with P < 0.05 were considered as significant. A regression analysis to test the association between lipid mediators and cellular infiltration was performed with Microsoft Excel 2010. R^2^ ≥ 0.5 were considered to indicate weak and R^2^ > 0.90 high correlation.

## Results

### In vivo study with healthy and diseased animals

To evaluate the effect of UfCP inhalation on the generation of endogenous lipid mediator in the lungs of healthy and allergic mice we performed a further analysis of the study of Alessandrini et al. [22].

**Figure 1:**
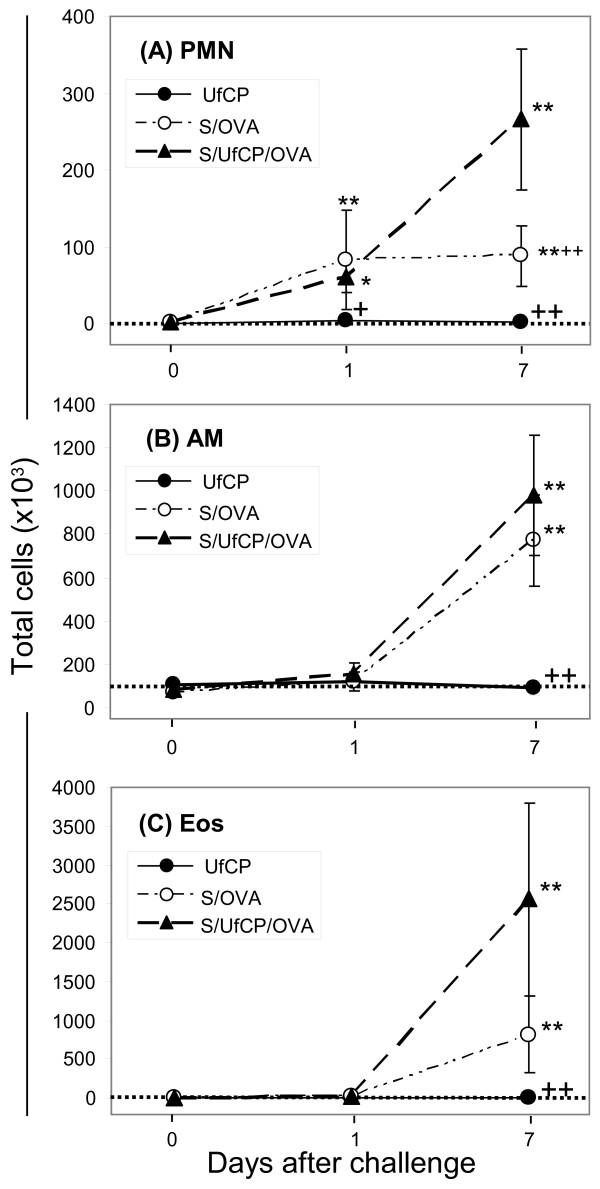
***In-vivo *study: BAL cell counts.** Total counts of neutrophils (**A**) alveolar macrophages (**B**) and eosinophils (**C**) retrieved by bronchoalveolar lavage in the UfCP, S/OVA and S/UfCP/OVA groups. Baseline levels which represent the data of the untreated group (1,6 ± 1,9 for neutrophils, 90,2 ± 25,6 for AM and 0 ± 0 for eosinophils) are shown by a dotted line. Data presented as mean ± SD (n = 4–9 per time points for BAL cells. *p < 0.05 and **p <0.01 vs baseline; ^+^p < 0.05 and ^++^p <0.01 vs S/UfCP/OVA).

In Figure 1 we present the total cell counts of neutrophils, alveolar macrophages and eosinophils, obtained by bronchoalveolar lavage after particle inhalation for each group on each time point. In non-sensitized UfCP mice there was no significant increase in all cell types on all time points. However in the S/OVA group and the S/UfCP/OVA group neutrophils, AM and eosinophils increased significantly on day 7.

**Figure 2:**
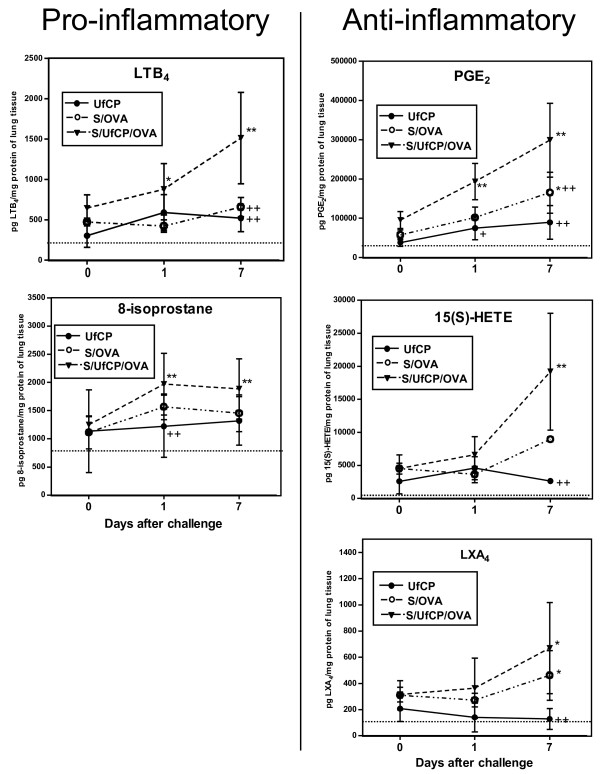
***In-vivo *study: Effect of UfCP inhalation on pro-inflammatory (left) and anti-inflammatory (right) endogenous lipid mediator levels in lung tissue of non sensitized and sensitized mice.** Non sensitized mice were exposed to UfCP for 24 h (UfCP group) or kept in filtered air (untreated group). OVA sensitized mice were exposed either to filtered air for 24 h and subsequently challenged with OVA (S/OVA group), or to UfCP for 24 h and then challenged with OVA (S/UfCP/OVA group). At day 0, 1 and 7 after particle exposure/OVA challenge the lungs were excised after BAL and used for analysis. The results are expressed in pg lipid mediator/mg protein analysed by specific enzyme immunoassays: Left panel: LTB_4_ and 8-isoprostane (8-isoprostane reprinted with permission from 22); right panel: PGE_2_, 15(S)-HETE and LXA_4_. Baseline values of untreated mice (dotted lines) are for LTB_4_: 264 ± 34 pg/mg; 8-isoprostane: 845 ± 231 pg/mg; PGE_2_: 34284 ± 4628 pg/mg; 15(S)-HETE: 1699 ± 189 pg/mg; LXA_4_: 125 ± 54 pg/mg. The data are given as absolute values (mean ± SD), n = 4-9/time point; *p <0.05 and **p <0.01 vs baseline; ^+^p <0.05 and ^++^p <0.01 vs S/UfCP/OVA.

Figure 2 describes the progression of each lipid mediator with a pro-inflammatory (left) or with an anti-inflammatory activity (right) on day 0, 1 and 7 after particle exposure in non sensitized and sensitized mice. On the pro-inflammatory side (Figure 2, left panel), compared to baseline (untreated group), the levels of LTB_4_ in the UfCP and S/OVA groups were elevated, although non significantly, 2-3-fold at all time points after particle inhalation. In the S/UfCP/OVA group after particle exposure followed by OVA challenge the levels of LTB_4_ significantly increased at day 1 (p < 0.05 vs baseline) reaching a 6-fold increase on day 7 (p < 0.01 vs baseline). At this time point the increase in LTB_4_ in S/UfCP/OVA was significant also compared to S/OVA and UfCP (p < 0.01). In order to complete the set of data of pro-inflammatory lipid mediators, the levels of 8-isoprostane, a lipid mediator used as a marker for oxidative stress, were added in the present manuscript, although published previously [22]. Its levels were significantly elevated in S/UfCP/OVA 1 and 7 days after particle inhalation and OVA challenge (3-fold). At day 1 the difference between UfCP and S/UfCP/OVA was significant (p < 0.01).

**Figure 3:**
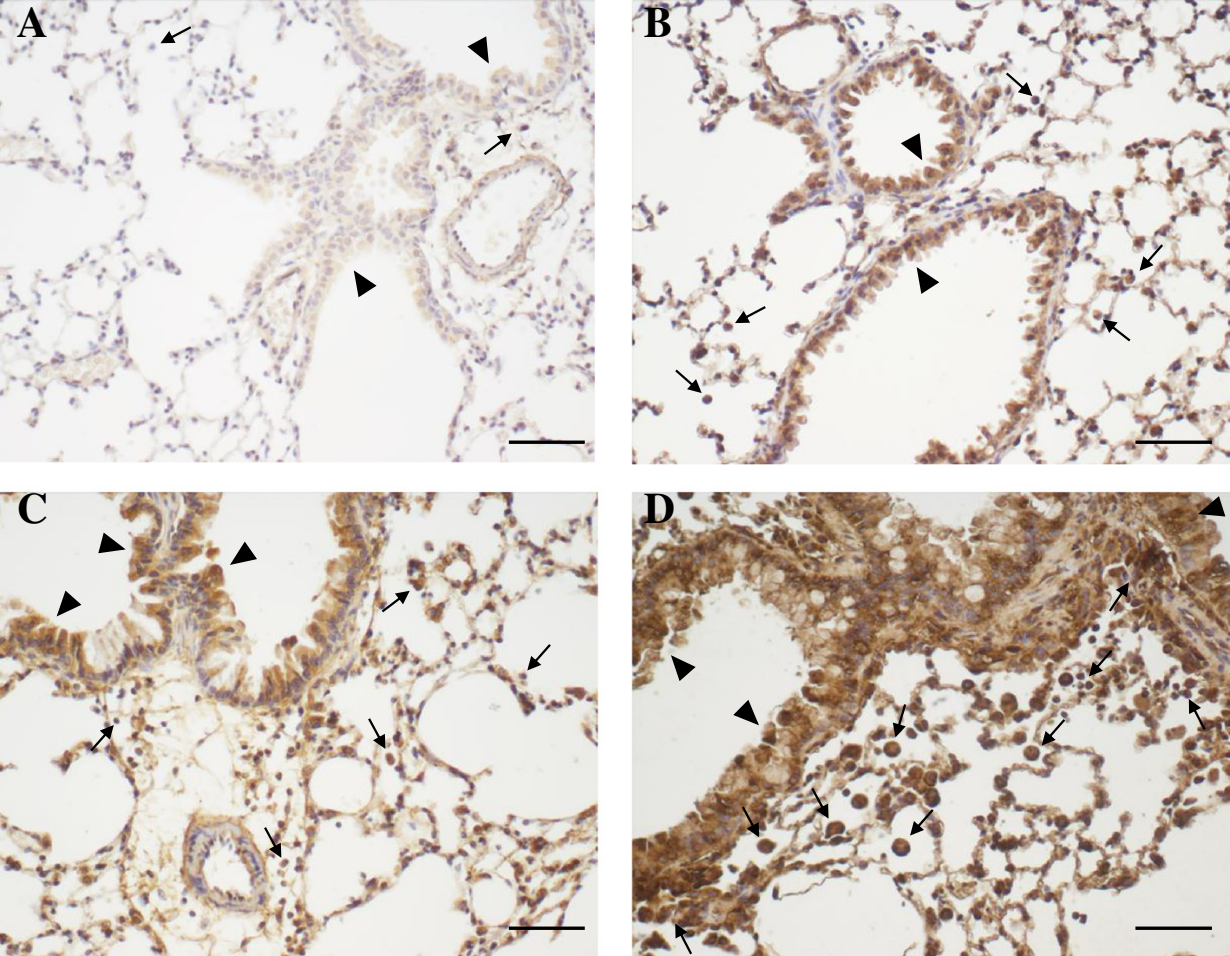
***In-vivo *study: Immunohistochemical analysis of 8-isoprostane in lung tissue.** Immunohistochemical analysis of 8-isoprostane in lung tissue was performed in formalin-fixed, paraffin-embedded mouse lungs from: (**A**) untreated mice, (**B**) non sensitized mice exposed to UfCP (UfCP group), (**C**) sensitized and challenged mice exposed to filtered air (S/OVA group), and (**D**) sensitized mice exposed to UfCP prior to OVA challenge (S/UfCP/OVA group), retrieved 7 days after UfCP exposure/OVA challenge. Arrowheads indicate bronchial and bronchiolar epithelial cells; arrows indicate inflammatory cells including alveolar macrophages. Scale bar, 50 μm.

**Figure 4:**
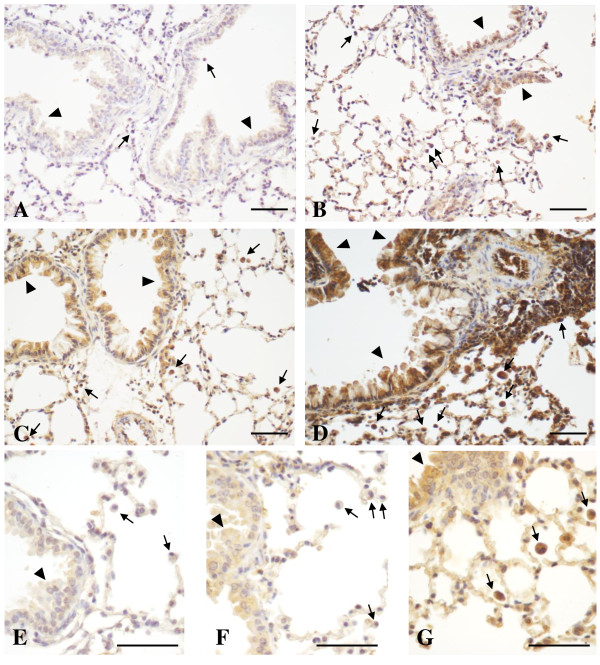
***In-vivo *study: Immunohistochemical analysis of PGE**_**2 **_**in lung tissue.** Immunohistochemical analysis of PGE_2_ in lung tissue was performed in formalin-fixed, paraffin-embedded mouse lungs from: (**A**) untreated mice, (**B**) non sensitized mice exposed to UfCP (UfCP group), (**C**) sensitized and challenged mice exposed to filtered air (S/OVA group), and (**D**) sensitized mice exposed to UfCP prior to OVA challenge (S/UfCP/OVA group) retrieved 7 days after UfCP exposure/OVA challenge. (**E**) untreated mice, (**F**) sensitized and challenged mice exposed to filtered air (S/OVA group), and (**G**) sensitized mice exposed to UfCP prior to OVA challenge (S/UfCP/OVA group) retrieved 1 day after UfCP exposure/OVA challenge. Arrowheads indicate bronchial and bronchiolar epithelial cells; arrows indicate inflammatory cells including alveolar macrophages. Scale bar, 50 μm.

Within the immune-modulating/anti-inflammatory lipid mediators (Figure 2, right panel), high PGE_2_ baseline levels in the lung of the untreated control group could be further enhanced, although not significantly, on day 1 and 7 after particle inhalation (up to 3-fold in UfCP vs baseline). In S/OVA the increase of PGE_2_ on day 7 was significant (p < 0.05 vs baseline, up to 5-fold). In S/UfCP/OVA the PGE_2_ level increased significantly to highest concentrations at day 1 and 7 (p < 0.01 vs baseline, 9-fold on day 7). PGE_2_ levels in S/UfCP/OVA were significantly increased compared to UfCP at day 1 (p < 0.05) and 7 (p < 0.01) and compared to S/OVA at day 7 (p < 0.01). The levels of 15(S)-HETE in UfCP were found to increase compared to baseline on day 1 after particle inhalation (3-fold), although not significantly. In S/OVA 15(S)-HETE was found to be elevated mainly on day 7 (up to 5-fold) and in S/UfCP/OVA at day 1 and 7, where only at day 7 reached statistical significance (p < 0.01 vs baseline and vs UfCP, up to 11-fold). Lastly, the levels of LXA_4_ in the UfCP group increased 2-fold straight after particle inhalation and decreased back to baseline levels thereafter. In S/OVA and S/UfCP/OVA the levels of LXA_4_ increased at all time points, but reached statistical significance only at day 7 (p < 0.05 vs baseline, with a 3- and 4-fold increase, respectively). In S/UfCP/OVA all mediators apart from 8-isoprostane significantly increased from day 0 to day 7 (p < 0.01). In order to test a possible association between lipid mediators and cellular infiltration, we performed regression analysis. We found a moderate correlation between neutrophil numbers and PGE_2_ (day 1 time point, R^2^ = 0.73), a weak correlation between alveolar macrophages and PGE_2_ (day 7 time point, R^2^ = 0.50) and also correlations between eosinophil numbers and LTB_4_, PGE_2_ and 15(S)-HETE (day 7 time points, R^2^ = 0.72, R^2^ = 0.78 and R^2^ = 0.59, respectively). With the aim of evaluating the localization of 8-isoprostane and PGE_2_ producing cells in the lungs of non sensitized and sensitized mice, we performed immunohistochemical analysis for 8-isoprostane and PGE_2_ in lung sections retrieved 7 days after particle/OVA inhalation (Figures 3 and 4A-D, respectively). Compared to the untreated controls with no detectable staining (Figures 3A, 4A), the UfCP group (Figures 3B, 4B) showed a weak staining for 8-isoprostane and for PGE_2_ in bronchial and bronchiolar epithelial cells and an inconsistent staining in inflammatory cells including AM (arrows), indicating a very mild inflammatory response accompanied by low levels of oxidative stress. In the S/OVA group (Figure 3C) 8-isoprostane staining was, similarly to UfCP group, present mostly in bronchial and bronchiolar epithelial cells (arrowheads) and in inflammatory cells including AM (arrows). The PGE_2_ staining (Figure 4C) was similar, but more pronounced compared to the same staining in the UfCP group. S/UfCP/OVA revealed the strongest 8-isoprostane and PGE_2_ staining localized in bronchial, bronchiolar (arrowheads) and alveolar epithelial cells and mainly in AM (arrows) (Figures 3D, 4D). No 8-isoprostane or PGE_2_ staining were detected where control IgG was used in the primary layer (data not shown).

Since the PGE_2_ levels in S/UfCP/OVA increased significantly already at day 1 time point, while in S/OVA remained close to UfCP (Figure 2, right panel), we performed an immunohistochemical analysis for PGE_2_ of both groups at day 1 time point. Our results show a strong difference between S/OVA and S/UfCP/OVA in the PGE_2_ staining especially in AM, where AM from S/UfCP/OVA stained more strongly compared to AM from S/OVA (Figure 4F and G, respectively). This indicates that AM are the first cells which drive the response after particle inhalation.

### In-vitro study with primary alveolar macrophages of healthy animals

**Figure 5:**
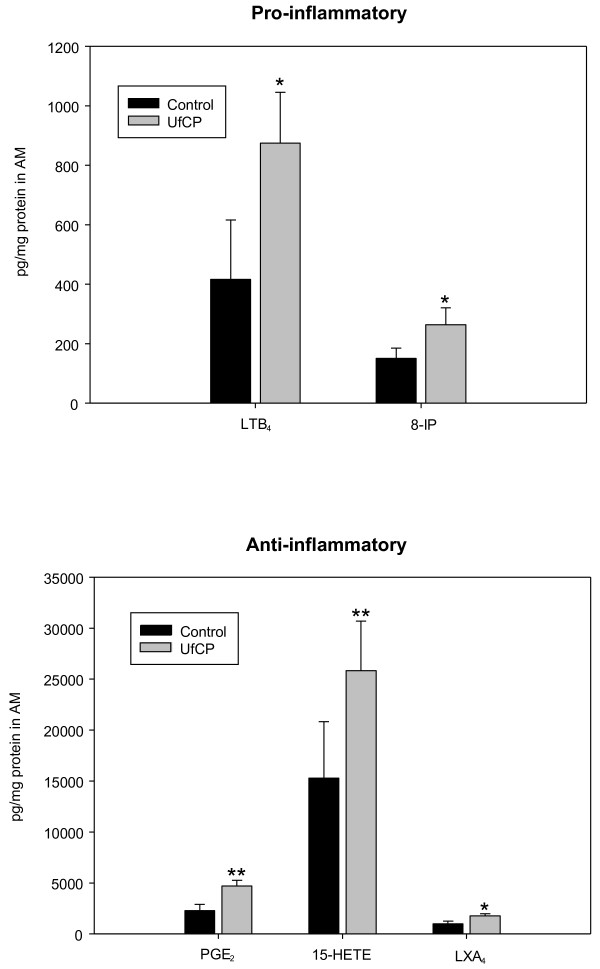
***In-vitro *study: Effect of UfCP on the generation of endogenous lipid mediator in primary rat alveolar macrophages.** Rat AM were incubated with UfCP (10 μg/1x10^6^ cells/ml) for 60 min (UfCP group). Control cells were incubated in parallel without particles. After incubation, the cells were immediately homogenised. The cell lysates were used for determination of the endogenous levels of pro-inflammatory (upper panel), and of anti-inflammatory (lower panel) lipid mediators. The data are given as absolute values (mean ± SD) with n as number of experiments (n = 4-6). *p <0.05 and **p <0.01 vs control.

Since in S/UfCP/OVA AM showed a strong staining for PGE_2_ already on day 1 after particle exposure (Figure 4G), we decided to evaluate the effect of UfCP on AM *in-vitro* and analysed the release of the same mediators as in the *in-vivo* study. As shown in Figure 5, UfCP induced a significant increase in the production of both pro-inflammatory (p < 0.05 vs control) and anti-inflammatory lipid mediators (p < 0.01 for PGE_2_ and 15(S)-HETE and p < 0.05 for LXA_4_) of about two-fold. The data were given as absolute values to compare the contribution of each lipid mediator in control and UfCP-treated AM and to the *in-vivo* results. The levels of PGE_2_ and especially 15(S)-HETE and LXA_4_ dominated the lipid mediator profile in the alveolar macrophages.

## Discussion

In the present study we investigated the effect of UfCP on the balance between the endogenous production of pro- and anti-inflammatory and pro-resolving lipid mediators in an *in-vivo* model with healthy and allergic mice and in an *in-vitro* model with AM from healthy animals. We chose UfCP since there is growing evidence that ultrafine ambient particles play an important role in the pathogenesis of respiratory diseases [14,15,28]. Primary ultrafine carbonaceous particles with a geometric diameter of 7 nm form chain-like aggregates similar to diesel exhaust particles [29,30]. Since the purpose of our study was to compare data relative to particle induced-synthesis of lipid mediators *in-vivo* and *in-vitro*, we have quantified the concentrations of lipid mediators in absolute values (pg mediator/mg protein) and we used similar particle concentrations/10^6^ cells in the two systems. In the *in-vitro* study we chose a particle concentration (10 μg/1 x 10^6^ AM/ml for 1 h incubation) which was optimal for eliciting production of lipid mediators in AM known from our previous findings [23,31]. For the *in-vivo* study, on the basis of a multiple path particle deposition model [32], we estimated a total particle mass deposition of 18.3 μg/mouse within 24 hrs using standard breathing conditions and assuming rat and mouse deposition probabilities to be similar. Considering adult mouse lungs to have a mean number of 2.9 (± 0.5) x 10^6^ AM [33], we can estimate an amount of 6.3 (± 1.1) μg UfCP/1 x 10^6^ AM in the lungs of a mouse for the 24 h in vivo exposure, not taking into account parameters like particle translocation or uptake by different cell types [34]. Similarly, in the *in-vitro* study, further parameters like cell-adherence, particle sedimentary and diffusive transport rates or particle coagulation rates in the liquid environment are not considered. In total, we only have reason for the assumption that exposure conditions in both studies might be comparable from the particle mass available per million of AM cells, which was 6.3 and 10 μg UfCP/10^6^ AM for the *in-vivo* and the *in-vitro* study, respectively. In spite of any drawbacks, this may be a first but significant hint for comparability.

**Figure 6:**
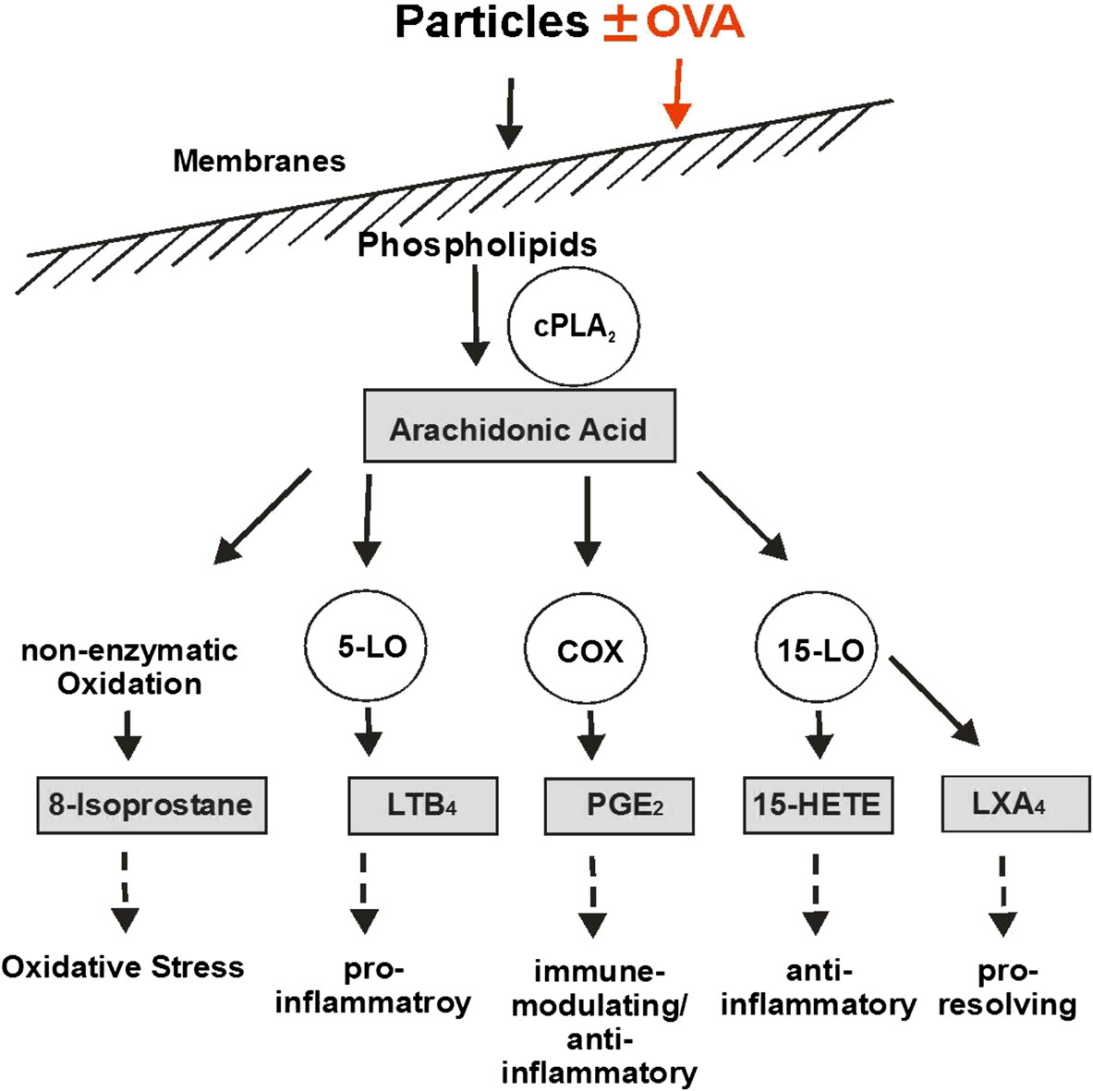
**Schematic representation of arachidonic acid-derived lipid mediators.** Particles and/or OVA challenge interfere with cell membranes to activate cytoplasmatic phospholipase A_2_ (cPLA_2_), which cleaves arachidonic acid from membrane phospholipids. Due to the oxidative potential of UFCP [23], arachidonic acid is non-enzymatically oxidized to 8-isoprostane, used as a marker for redox imbalance. Arachidonic acid serves as a substrate for the downstream pathways of COX (cyclooygenase), 15- and 5-LO (15- and 5-lipoxygenase), leading to formation of prostaglandins such as PGE_2_, 15(S)-HETE, leukotrienes such as LTB_4_ and to LXA_4_ via PGE_2_ activated 15-LO [23,26,35]. Because these processes are predominantly located in the nuclear membrane or perinuclear space, these products of these pathways may influence gene expression. LTB_4_ activates the inflammation, whereas PGE_2_, 15(S)-HETE and LXA4 are immune-modulating, anti-inflammatory and pro-resolving lipid mediators, respectively.

One limitation of our study is that we did not use AMs from control and sensitized mice for the *in-vitro* study; we preferred to use primary AM of healthy rats, because of the higher cellular yield in bronchoalveolar lavage of an individual rat compared to that of a mouse. UfCP are phagocytosed by AM and can be found in phagolysosomes of the cells [22,23,34]. The *in-vitro* study, these particles exerted a significant stimulating effect on all lipid mediators by about two-fold (Figure 5). Because of the different baseline levels of the single mediators, the involvement of each mediator contributed, depending on its concentration, to the outcome of the AM response by the particles. In the inflammatory reactions induced by the particles the properties of immune-modulating PGE_2_, anti-inflammatory 15(S)-HETE and pro-resolving LXA_4_ (Figure 5, lower panel) dominated the mediator profile compared to pro-inflammatory LTB_4_ and oxidative stress marker 8-isoprostane (Figure 5, upper panel) and could counterbalance the effect of LTB_4_ and oxidative stress. This is consistent with our previous *in-vitro* findings showing an inhibiting effect of UfCP-treated AM on the respiratory burst activity of stimulating neutrophils [31]. When particle-treated AM were pre-incubated with indomethacin (i.e. an inhibitor for cyclooxygenase and therefore also for PGE_2_ production), the down-regulating effect of the particles on the respiratory burst activity of stimulated neutrophils was abolished. Recently we have shown with primary canine and human AM that UfCP activated cytoplasmatic phospholipase A_2_ (cPLA_2_), the initial enzyme for the production of lipid mediators, liberating arachidonic acid from membrane phospholipids (see Figure 6; [23]). We also observed this in rat AM (data not shown). Moreover we showed that arachidonic acid was metabolised to the different mediators by cyclooxygenase to PGE_2_, by 15-lipoxygenase to 15(S)-HETE [25], and by 5-lipoxygenase to LTB_4_. In addition arachidonic acid was oxidized to 8-isoprostane by non-enzymatic oxidation indicating that the particles induced oxidative stress reactions in the cells. This was due to the oxidative potential of the particles; we found that particles were able to oxidise the amino acid methionine to methionine sulfoxide, and that they showed an ESR signal equivalent to a free radical within the particle core [23]. The pro-resolving mediator LXA_4_ also increased during particle incubation, which is in agreement with our recent findings with human neutrophils [35]. LXA_4_ is generated via PGE_2_ and 15(S)-HETE and represents a stop signal for inflammation to return the inflamed tissue to homeostasis [36].

In non-sensitized mice, inhalation of UfCP alone induced a light, non significant increase of all mediators, apart from LXA_4_ (Figure 2). LTB_4_ is a potent chemoattractant for neutrophils [37], but the increase of LTB_4_ following particle exposure alone did not provoke an influx of neutrophils (Figure 1). Obviously, the particle-induced production of LTB_4_ seems to be too low to induce a neutrophil influx into the lungs. PGE_2_ and 15-HETE showed higher baseline levels compared to the other mediators, as previously described for PGE_2_[2] and consequently showed the highest production after particle exposure. We have previously shown that particle exposure induces a transient and moderate NF-κB activation and TNF-α expression [22,38]. We can speculate that healthy mice respond to particle exposure with a very light and transient inflammation due to the dominating endogenous levels of immune-modulating PGE_2_ and anti-inflammatory 15(S)-HETE, probably supporting the suppression of pro-inflammatory mediators including LTB_4_. Our *in-vivo* and *in-vitro* findings show a similar immune-modulating/anti-inflammatory response to particle exposure in AM from healthy rats and in lung tissue from healthy mice.

In sensitized mice, exposure to allergen challenge alone (S/OVA group) induced a significant increase of PGE_2_ and LXA_4_ compared to untreated control (Figure 2). Although the endogenous levels of PGE_2_ and 15(S)-HETE (up to 5-fold on day 7) again dominated the mediator profile, they were not able to counterbalance the allergen-induced inflammation. The analysis of the lipid mediators in sensitized animals exposed to UfCP inhalation only (without OVA challenge) was not performed since both at the functional and at the BAL cell infiltration level the response was not different compared to NS/UfCP [21,22]. In S/UfCP/OVA, exposure to UfCP before allergen challenge increased significantly the levels of all mediators. These data were confirmed by our immunohistological studies showing increased staining for 8-isoprostane and for PGE_2_ in S/OVA (Figures 3C and 4C) compared to healthy controls, which increased further in S/UfCP/OVA (Figure 3D for 8-isoprostane and Figure 4D for PGE_2_). In addition, the immunohistochemical analysis of PGE2 on day 1 revealed a very strong staining for PGE2 mainly in AM of S/UfCP/OVA vs S/OVA (Figure 4E to G). We therefore suggest, that AM are the first cells driving the force for the particle-induced early inflammatory responses.

PGE_2_ is known as an immune-modulator and exerts its action by binding to one or to a combination of its four subtypes of receptors EP_1_, EP2, EP3 and EP4 [39]. The EP receptors are expressed on the nuclear membranes of cells. They are functional due to modulation of gene transcription providing an additional role of PGE_2_ in the regulation of cell functions [40]. Furthermore, cyclooxygenase-2 derived prostaglandins, including PGE_2_, are not only involved in the onset of inflammation but serve also an important role in the resolution of inflammation. This is due to the ability of PGE_2_ to induce 15-lipoxygenase expression for production of 15(S)-HETE thus promoting the biosynthesis of lipoxins such as LXA_4_[41,42]. In a model of acute lung injury, selective COX-2 inhibition or deficiency results in prolonged inflammation by decreasing production of PGE_2_ and pro-resolving mediators including LXA_4_[41,43]. We have recently reported that PGE_2_ is able to modulate iron oxide particle-induced inflammation in a model of healthy rat AM *in-vitro*. Depending on size and surface area per mass, small iron oxide particles (diameter: 0.5 μm; specific surface area: 17 m^2^/g) induced a high endogenous level of PGE_2_ in AM which suppressed the release of the pro-inflammatory cytokine IL-6 [25]. In contrast, larger iron oxide particles (diameter: 1.5 μm; specific surface area: 7.1 m^2^/g) induced only a moderate endogenous level of PGE_2_ which was not able to prevent the IL-6 release. In a parallel conducted *in-vivo* study with healthy rats the larger particles caused an inflammation in the lung, whereas the smaller particles did not [25]. These findings underline the immune-modulating and anti-inflammatory properties of PGE_2_. The relative high concentrations of PGE_2_ released in S/UfCP/OVA were not able to counteract the allergic inflammatory response. An interesting paper from Martin et al. [44] reported for a rat model of allergic asthma that external PGE_2_, administered in concentrations of 1 to 3 μg by intratracheal insufflation 30 min before OVA challenge, inhibited the early and late response of pulmonary resistance and reduced the bronchoalveolar levels of eosinophils and cysteinyl-leukotrienes. The authors concluded that PGE_2_ is a potent inhibitor of allergic airway responses in a rat model of allergic asthma. However, in our study we measured the endogenous levels of all lipid mediators. Concerning the endogenous PGE_2_ levels at day 7, S/OVA showed with 0.165 μg PGE_2_/mg protein an about 20 times lower level and S/UfCP/OVA with 0.3 μg PGE_2_/mg protein a 10 times lower level than the externally added PGE_2_ concentrations (1 to 3 μg) in the study of Martin et al. [44]. We therefore suggest that the endogenous levels of immune-modulating PGE_2_ and anti-inflammatory 15(S)-HETE were not high enough to counterbalance the endogenous levels of pro-inflammatory LTB_4_ and other inflammatory mediators during the allergic inflammation.

Also the pro-resolving mediator LXA_4_ was unable to contribute to a reduction of the severe inflammatory response in the S/OVA and S/UfCP/OVA groups probably because of its low endogenous levels (Figure 2, right panel). On the contrary, high endogenous levels of LTB_4_ in S/UfCP/OVA were accompanied by a strong increase of neutrophils, although the regression analysis at single animal level for this association in all experimental groups did not have enough power to detect a correlation (R^2^ < 0.25), (Figures 1 and 2). The inflammatory response was accompanied by an increase in oxidative stress as indicated by the increase in 8-isoprostane determined by its specific analysis and by immunohistochemistry. The inflammation was probably supported by the high amounts of eosinophils in the lungs of S/OVA, which increased even more by the particle exposure in S/UfCP/OVA, as shown in Figure 1. Eosinophils contribute to allergen-induced lung inflammation by producing cysteinyl leukotrienes, derived via activation of 5-lipoxygenase, and eosinophil cationic protein [45-47]. Furthermore, it is known from the blood of patients with severe asthma that decreased levels of LXA_4_ are coexisting with increased ones of LTB_4_ and cysteinyl leukotriens [48]. Interestingly, we obtained a strong correlation (R^2^ > 0.7) between BAL eosinophils and LTB_4_ or PGE_2_ levels in the lungs. Recently it was shown, that AM of patients with non-severe and severe asthma contained lower levels of LXA_4_ than those of normal individuals resulting in a pro-inflammatory imbalance [49]. Moreover, the airway levels of LXA_4_ and the expression of its biosynthetic enzymes, including cyclooxygenase-2 and 15-lipoxygenase, and the LXA_4_ receptor were markedly decreased in severe asthma [50]. We did not observe a decrease in anti-inflammatory mediators following allergen challenge probably because our mouse model is characterized by mild allergic inflammation and lacks the changes in the inflammatory milieu that are described in severe asthma models.

In a recent study in our mouse allergy model, inhalation of ultrafine carbon particles prior to OVA challenge caused most significant changes in Clara cell protein CC16 in BALF and serum, BALFs total protein and TNF-α expression in lung homogenates and the strongest morphological alterations of Clara cells and goblet cell metaplasia in sensitized mice compared to non-sensitized mice [38]. The data suggest that in the allergic mice the mechanisms to compensate the alterations of Clara cells and globlet cell metaplasia due to particle exposure are insufficient [38]. All these observations support the concept of an insufficient role of the anti-inflammatory lipid mediators in counterbalancing the strong inflammatory responses.

## Conclusion

Particle exposure increased the release of endogenous lipid mediators, and affected their pro- and anti-inflammatory balance, both in our *in-vivo* and *in-vitro* studies. In AM and lung tissue of healthy animals immune-modulating/anti-inflammatory PGE_2_, anti-inflammatory 15(S)-HETE and pro-resolving LXA_4_ dominated the lipid mediator profile and thus contribute to dampen the already mild inflammatory response caused by particle exposure. On the contrary, the exposure of allergic mice to particle inhalation (S/UfCP/OVA) aggravated further the already ongoing inflammation of the allergic mice, shifting the endogenous balance strongly to pro-inflammatory responses. Although PGE_2_ and 15(S)-HETE were still present in high amounts, their endogenous levels were not sufficient to counterbalance the inflammation. Since UfCP represent the core of diesel exhaust particles [29,30], our data suggest an aggravation of allergen-induced lung inflammation by environmental particles.

## Abbreviations

UfCP: Ultrafine carbon particles; AM: Alveolar macrophages; LTB_4_: Leukotriene B_4_; PGE_2_: Prostaglandin E_2_; 15(S)-HETE: 15(S)-hydroxy-eicosatetraenoic acid; LXA_4_: Lipoxin A_4_; OVA: Ovalbumin; BAL: Bronchoalveolar lavage.

## Competing interests

The authors declare that they have no competing interests.

## Authors’ contributions

IBS and FA conceived the overall research idea, made substantial contributions to acquisition, analysis and interpretation of data and wrote the manuscript. EK supervised particles exposures. HB and TS significantly helped in the revision of the manuscript. All authors read and approved the final manuscript.

## In memoriam Dr. Ingrid Beck-Speier

**Figure 7:**
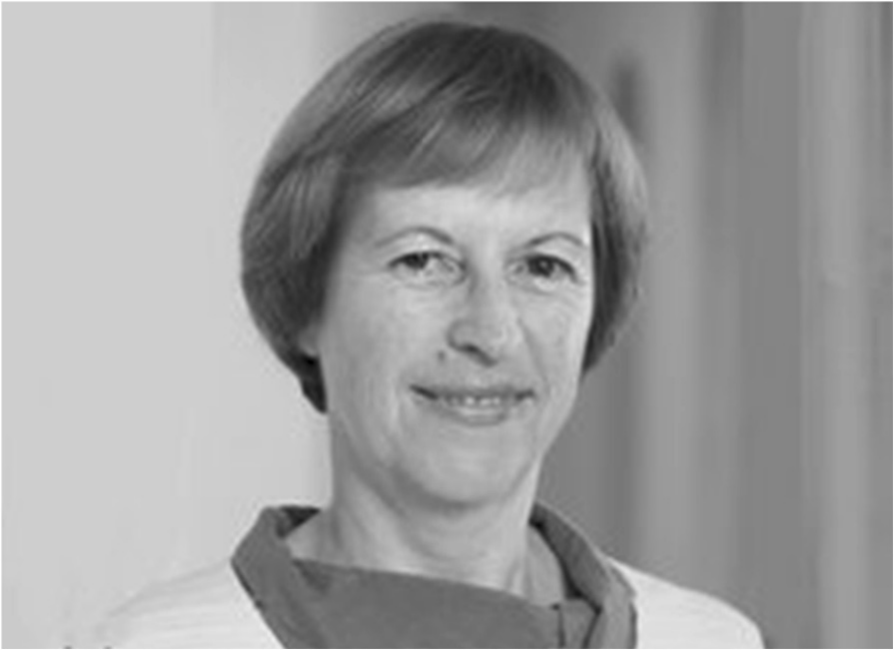


On the 3^rd^ of April 2011, Ingrid Beck-Speier (Figure 7), employee of the Comprehensive Pneumology Center, Institute of Lung Biology and Disease of the HelmholtzZentrum München, Germany, died after a long-term disease.

She was a renowned scientist who especially built up her reputation by contributing to the understanding of the impact of the particle surface on the inflammatory effect of ultrafine particles or nanoparticles.

After achieving her PhD in chemistry at the Albert-Ludwigs-University in Freiburg, in 1979 she joined the department for Enzyme Chemistry of the HelmholtzZentrum München, formerly named GSF-Research Center. Her work focused on cell communication, oxidative metabolism of phagocytes and, very dedicatedly during the last years, on the synthesis of lipid mediators induced by oxidative stress.

Beside her enthusiasm for science, Ingrid Beck-Speier had a special love for arts and active painting. Her love for nature and her outstanding friendliness to all her co-workers and friends is shown in her colorful way of interpretation of blooming meadows.

We will always keep her in our thankful memory.
